# The Master’s Tools—Anti-Bullying and Harassment Policy in Higher Education Institutions

**DOI:** 10.3390/ijerph23060706

**Published:** 2026-05-26

**Authors:** Margaret Hodgins, Carol Ballantine, Patricia Mannix McNamara

**Affiliations:** 1 Health Promotion, School of Allied and Community Health, College of Medicine, Nursing and Health Sciences, University of Galway, H91TK33 Galway, Ireland; 2 School of Geography, University College Dublin, D04F6X4 Dublin, Ireland; carol.ballantine@ucd.ie; 3 School of Education, University of Limerick, V94T9PX Limerick, Ireland; 4 Human Rights, Equality Diversity and Inclusion, University of Limerick, V94T9PX Limerick, Ireland

**Keywords:** anti-bullying policy, sexual harassment policy, prevention, health, health gain

## Abstract

**Highlights:**

**Public health relevance—How does this work relate to a public health issue?**

- This paper addresses gender-based violence and harassment, a serious public health issue and a leading cause of physical and mental illness.
- Understanding how determinants of health, such as educational and workplace settings, exert their influence on health is essential to protecting and improving the health of the public.

**Public health significance—Why is this work of significance to public health?**

- The pervasiveness of gender-based violence and harassment in higher education renders this setting potentially damaging to health for both students and staff.
- High levels of gender-based violence and harassment are facilitated by the non-performativity of policies, which potentially inflicts further damage to targets or victim-survivors.

**Public health implications—What are the key implications or messages for practitioners, policy makers and/or researchers in public health?**

- Higher education institutions, specifically those who are tasked with dealing with gender-based violence and harassment, need to move beyond legalistic and bureaucratic approaches, focusing on the problem as a public and occupational health issue rather than an individualistic framing of ‘problem’ individuals and the presence or absence of ‘problem’ behaviours.
- Restorative justice approaches which focus on who has been harmed, what their needs are and how they can be addressed merit serious consideration.

**Abstract:**

The persistently high prevalence of gender-based violence and harassment (GBVH) in higher education institutions is a well established phenomenon, as is the inadequacy of institutional responses and the silencing of those who aim or attempt to report it. Drawing on Ahmed’s concept of ‘non-performativity’, ‘institutional speech acts that do not bring into effect what they name’, this paper argues that the non-performativity of anti-bullying and harassment policy is an exercise of power, consistent with Agócs concept of institutionalised resistance. Reporting misconduct is intentionally transformational, but seen as a threat to powerful organisational actors, who exercise institutional power to enact procedures in such a way that victim-survivors are unvoiced and tricked into ‘reluctant acquiescence’ with adverse consequences on their personal and occupational health. We employ documentary analysis to critique policies and procedures for GBVH in Irish universities, and specifically how institutional power is exercised through policy documents. The analysis is based on ten pseudonymised universities, rendering a sample size of 23 documents, pertaining to GBVH for staff. We find that the tone and language employed in policies, and the way in which the informal and formal approaches in anti-bullying and harassment policies frame the problem, serve the interests of the institution. Confidentiality clauses, the framing of the problem as an individualistic, incident-based problem, to be resolved case-by-case, and quasi-legal processes facilitate non-performativity, preserving institutional power and the status quo. From a public health perspective such inertia undermines efforts to prevent harm and promote workplace wellbeing. Meaningful reform will require that HEIs employ alternative tools capable of unsettling these entrenched institutional arrangements and to adopt alternative, proactive tools that prioritise accountability, transparency, prevention and health gain. We suggest new tools in the form of victim-centred, trauma-informed, remediation- and restorative-based approaches.

## 1. Introduction

Bullying behaviour, sexual harassment and everyday sexism are widespread, reinforcing cultural values that celebrate aggression and hypermasculinity [1]. This is reflected in the pervasiveness and normalisation of gender-based bullying, violence and sexual harassment (GBVH) within organisations and, in particular, higher education institutions (HEIs) [2,3,4,5,6,7]. The deleterious impact of these behaviours is well documented, including anxiety, depression, burnout, post-traumatic stress disorders and cardiovascular illness [8,9,10], rendering this phenomenon significant from a public health perspective.

Equally well established is the inadequacy of institutional responses and the silencing of those who aim or attempt to report it [11,12,13,14,15]. Institutional responses are mediated through policies, environmental structures that guide organisational action, and, in this context, impact on health and wellbeing outcomes. From a public health perspective, workplace conditions are central to health gain, as emphasised in the Ottawa Charter for Health Promotion, which highlights that health is created within the settings of everyday life; consequently, the design and implementation of complaint responsiveness and outcomes can either enable or constrain equitable improvements in employee wellbeing [16]. Recent scholarship identifies the wilful blindness, that is ‘the conscious avoidance of knowledge or information to evade responsibility’ [17], that is used to avoid addressing gender-based violence and harassment. This malevolent circularity, where the harm caused by experiencing GBVH is intensified and exacerbated by the failure to protect and remediate the victim-survivor [18], is clearly damaging to health. Further, it has the effect of reproducing the power inequalities and dependency relations that characterise academia.

In her text *Complaint!*, Sara Ahmed, quoting Audre Lorde, advocates that ‘*the master’s tools will never dismantle the master’s house*’ [19]. Ahmed argues that the processes and procedures for addressing GBVH in universities that act to suppress or silence those who complain permit perpetrators to act with impunity, and are best described as ‘non-performativity’, ‘*institutional speech acts that do not bring into effect what they name*’ [19]. Building on the work of O’Connor et al. [20] on the need to understand power in order to understand GBVH in higher educational institutions (HEIs), we argue that the ‘non-performativity’ of anti-bullying and harassment policy is in itself an exercise of power, consistent with Agócs concept of institutionalised resistance [21]. In agreement with Ahmed’s position that reporting misconduct is intentionally transformational [19], we also argue that it is an opportunity for healing, repair and recovery (health gain) but that in reality it is conversely seen as a threat to hierarchy and powerful organisational actors who enact procedures in such a way that victim-survivors are unvoiced and tricked into ‘reluctant acquiescence’ [13,22].

Policies are the most frequently employed and most commonly recommended tools to address bullying and harassment, yet they remain an under-researched aspect of workplace bullying [23] and of GBVH in universities [24]. Policies are part of the organisational environment and are constitutive of the problem. Specifically, although gendering practices and trends such as criminal justice drift have been explored (see for example [12,25,26,27]), the way in which policy documents are formulated, framed and implemented has not yielded effective impact; rather, we argue that they can function as a deterrent to action, undermining health gain and, in some cases, contributing to adverse health outcomes by perpetuating inaction and reinforcing existing disempowering inequities.

We employ documentary analysis to critique staff policies and procedures for GBVH in Irish universities. Specifically, we explore how institutional power is exercised through policy documents. Although GBVH policies explicitly aim to prevent bullying and harassment, they frequently fail to do so, and in a subtle inversion, they conversely serve to uphold the ‘House of the Master’.

## 2. Gender-Based Harassment and Violence (GBVH) in Higher Education

Understanding the determinants of health, including health inequity, is essential to protecting and improving the health of the public. Educational opportunities and working conditions, both intrinsic to the higher education setting, exert powerful effects on health. Health (or ill health) is created in the nexus between individuals, organisational structures and organisational processes [16]. Despite being potentially enriching environments, the pervasiveness of violence in the form of bullying and harassment, which has been described as epidemic in academia [22], renders this setting potentially damaging to health gain for both students and staff. HEIs are in a unique position to influence social and cultural development in the context of addressing violence throughout society [28]. According to Tsouros (p. 11) universities committed to the principles of health for all and sustainable development can be a tremendous asset to their staff and students, to the communities in which they are located and to the wider society where their students and trainees will eventually assume professional roles [29]. They not only shape the social and environmental conditions of staff and students, but they also act as key sites for health promotion; therefore, ineffective policies in areas such as bullying and gender-based violence and harassment risk undermine both individual wellbeing and broader population health. Universities as the sites of education of those who will work in public health have a role to play in modelling good organisational practice and ‘decent work’ [30].

In keeping with the approach taken in both the ILO first global survey of experiences of violence and harassment at work [30] and the UniSAFE survey of 46 universities across 15 countries in Europe [31], we have adopted the nomenclature of gender-based harassment and violence. We use the term to refer to a multi-faceted construct embracing ‘*incidents that disproportionately affect people because of their sex, gender, or sexual orientation (including being female, being a woman, not conforming to hegemonic masculinity ideals, being non-binary, not being heterosexual, being trans, being queer, etc.) based on exploiting and reifying gender power relations; and feeds from prejudiced and/or discriminatory norms, attitudes and stereotypes in the wider environment*’ [32] (p. 1692). We favour the concept of *violence* over the more frequently used bullying, consistent with the notion of violence in workplaces as a continuum [33,34]. Violence can be physical, sexual, psychological and economic, instrumental or symbolic [35,36], and can have varying degrees of severity and expressions (online, work-related and face-to-face). We retain the term *harassment* to ensure due attention to sexual harassment, which is often excluded in studies of workplace bullying. Although victims of violence in HEIs can be of any gender, we use ‘*gender-based*’ given the known gender inequalities in higher education, which mutually support and reinforce sexual harassment [37] and which intersect with other bases of inequality such as race, ethnicity, disability and identity [32]. Not just women but any person who does not conform to a heteronormative or hypermasculine identity are at increased risk for harassment [38]. While there are variations in the experiences of GBVH, all have power abuse as a common root, whether that takes the form of hierarchical, social or emotional power. In the context of higher education as a setting where power is exercised across multiple fora, and where senior academics can appear to be inviolable, a function of the unique nexus of reward, positional, informational, and referent power in the HE context [9] is the HE setting particularly, potentially, dangerous.

### 2.1. Prevalence

Prevalence estimates are complicated by the variation in measurement approach, yet, methodological inconsistencies notwithstanding, the rates are indicative of a serious problem in the sector. Indeed, bullying and harassment have been described as epidemic in academia [22] and gender-based violence as a major and urgent issue across HEIs and RPOS [39]. Manuel et al. (2023) citing Mahmoudi and Keashley (2021) identify that the negative personal and psychological effects of experiencing or witnessing academic bullying are extensive, with symptoms including anxiety, depression, emotional exhaustion, and burnout [40,41]. The recent UniSAFE behavioural survey of gender-based violence involving over 42,000 individuals across 43 participating European universities and research producing organisations (RPOs) found an overall rate of any form of gender-based violence since they started working in the institution to be 73.7%. Within this, 70.3% experienced psychological violence, 35.5% sexual harassment and 1.2% sexual violence [42,43]. Non-binary and female respondents experienced higher levels of physical, economic, sexual and psychological violence [42]. Experiences were summarised overall as follows: ‘*Regardless of when it happened, the experiences show a very similar pattern; they happened more than once and were often frequently repeated, lasting for months and years and demonstrating an interplay of multiple forms of gender-based violence*’ [43] (p. 217). With regard to HEIs in Ireland, national studies find workplace bullying rates of 28% and 33.5% (depending on whether a behavioural checklist or self-labelling method was employed), experienced in the past three years [7], while 21–35% of staff in Irish HEIs experienced some form of sexual harassment in the past four years [5]. Minority groups were more likely to experience bullying compared to majority groups (i.e., heterosexuals, ethnic majority groups and respondents with no disabilities) [7] while women and non-binary respondents experience higher levels of exposure to sexualised comments, sexual hostility, unwanted sexual attention, and some aspects of sexual coercion and violence [5].

Consistently higher levels of exposure for women, ethnic minorities and non-binary staff [42,44,45] underscore the role of power and of structural inequalities, and highlight the reality that any study of GBVH is de facto a study of wider and organisational power relations, and, as Humbert et al. (2025) argue, there is a need for analyses to be intersectional and contextual [32]. High prevalence rates are unsurprising in the context of a neo-liberalised sector, in which particularly unequal power and dependency relations, hyper-competitiveness, performativity and precarity are common organisational features [14,20,46] creating a structural and cultural climate that both increases the risk of various forms of violence and mitigates attempts to tackle them with significant implications for health. In particular, such environments can normalise chronic stress, psychological distress, and burnout, embedding ill health as an ordinary feature of academic life rather than an exceptional outcome requiring intervention.

### 2.2. Impact

The deleterious impact of GBVH on individual health and wellbeing is unequivocal and HEIs are no exception to this general finding. Almost all (95.9%) of respondents in the UniSAFE survey experienced illness as a result of gender-based violence, while 40% felt unsafe and 70% felt socially excluded [42]. From a public health perspective, these findings highlight gender-based violence as not only a social and institutional issue but also as a determinant of physical health, mental health, and emotional health with population-level implications for wellbeing, participation, and health equity within higher education settings.

Bondestam and Lundqvist identify a range of adverse consequences including sexually transmitted diseases, unwanted pregnancy and alcohol abuse from their systematic review of sexual harassment in HEIs [2], as well the more frequently reported mental health difficulties, such as anxiety, depression, loss of confidence, feelings of powerlessness and being ‘worn down’ [2,7,9,47]. The increased risk of GBVH for minoritized groups underscores the role of GBVH in HEIs as a determinant of health inequity. Opportunities for promotion, funding and collaboration are affected, while for some, withdrawal from the organisation is the only option [2,6,43,48]. The wider public health concerns are intensified with discrimination and work-related exclusion [49], decreased engagement and lowered self-rated performance [50] reflected in significant economic costs [51]. The severe negative impact of non-response or poor response to reporting GBVH has received research attention more recently, with evidence of career and financial impact [52,53], shame [54] and re-traumatisation [9].

### 2.3. Legal and Policy Context

With regard to the legal context, the Irish legislative framework includes health and safety, criminal justice domestic violence and employment equality ordinance, although none are specific to HEIs (a comprehensive outline of the legislative framework can be found in the UNIsafe national fieldwork report Shinkwin (2021)) [28]. Ireland has ratified the Istanbul Convention (Council of Europe Convention on Preventing and Combating Violence Against Women and Domestic Violence) and since 2016 has made concerted efforts to tackle GBVH in higher education, as evidenced by the *Safe, Respectful, Supportive and Positive; Ending Sexual Violence and Harassment in Irish Higher Education Institutions* Framework (ESVH) and the commissioning of national prevalence surveys (e.g., [5,7,55]) building on a Prevention, Protection, Provision of services, and Policy coordination model. This has been followed by an implementation plan focusing on reporting, supports and the campus environment under the auspices of the statutory planning and development body for higher education (Higher Education Authority) [28]. More generally, while Irish HEIs were initially slow to come to the riverbank of neoliberal ideology, they are now fully immersed in the flow [56], itself associated with aggression, hyper-competitiveness and institutional violence [57]; the sector has also been the subject of media and academic attention with regard to gender inequity, itself a driver and consequence of GBVH [37]. A National Gender Equality Taskforce and Gender Action Plan, along with annual monitoring and the requirement of an Athena Swan charter as a condition of future funding [28], have highlighted environmental factors including micropolitical processes and practices [20]. While these developments have been associated with significant yet small improvements in gender balance for staff groups, progress is slow [58] and is very much driven by persistent adherence to address issues using the ‘master’s tools’ (for a thorough analysis of these issues in the Irish HE sector, see O’Connor, P., White, K. (2021) *Gender, Power and Higher Education in a Globalised World*, Palgrave Macmillan).

## 3. Institutional Response and Silencing

Despite the significant individual and institutional impact of GBVH, the response within HEIs, avowed egalitarian institutions, appears to be distressingly inadequate to the point of perfidy [9,25,27,52]. Consistent with the emphasis within public health on prevention, the institutional response encompasses anti-bullying/GBVH/harassment/sexual misconduct redress policy, including information and education and wider supports for victim-survivors who have experienced compromised health. In the context of HEIs, poor response and weak remediation have been variously referred to as institutional inaction [59], institutional blindness [60], and institutional betrayal [27,61]. This deficiency is supported by a range of overlapping strands of evidence, in particular survey responses about what actions (if any) victim-survivors take and analysis of experiences of organisational response through qualitative studies.

A review of studies of bullying in the HE sector found that 57% of victim-survivors told their supervisor or Dean, 27% told HR and only 16% made a formal complaint. Of note, their actions were more likely to make the situation worse, rather than better. None of the strategies changed the situation for a sizable proportion of victim-survivors [62]. A systematic review of sexual harassment in HE concluded that only a small proportion of all sexual harassment is reported formally, as is also the case for other types of sexual violence; for example, only 12% of victim-survivors filed a formal report [2,63]. By far the most comprehensive study of policy has been undertaken in the UniSAFE project, which reported on 16 case studies (where policy, training, support, etc., were present) of institutional response to gender-based violence across 48 RPOs. In terms of coverage in the HE sector, Huck et al. (2022) identified that only 35% of their sampled universities and RPOs had policies, protocols, action plans, leaflets, or informational documents that focused solely on combating gender-based violence in their institution [64]. In general, an implementation gap between what was on paper and what was actually implemented was observed. Such gaps or disconnects can weaken protective structures, delay or prevent effective intervention, and ultimately exacerbate adverse health outcomes, as intended safeguards fail to translate into meaningful improvements in safety, wellbeing, and equity. Most institutional responses did not include evaluation mechanisms and relied on informal procedures as well as formal, with institutional resistance present in the form of insufficient allocation of resources. There was a tendency to approach the problem as an individual issue and correspondingly an incident-driven approach was adopted, which was limiting in terms of confronting the structural factors behind violence. Relatedly, power relations and hierarchical structures were the strongest hindering factors, with robust hierarchies and academic dynamics possibly preventing disclosure and reporting [60]. Framing harm as an individual issue aligns well with an incident-driven approach, in which responses are activated only after specific events occur and are individualised (often accompanied with a blame approach such as the common personality clash response). Reactive models limit opportunities for prevention, precisely because they overlook the structural and environmental conditions that produce risk in the first place, thereby reducing the potential of health protection and health promotion.

### Lived Experience

In terms of the lived experience, several studies attest to very negative experiences of institutional response, reinforcing the general failure of HEIs to address GBVH in a meaningful, effective manner. Unclear procedures, the reframing and individualising of the situation and active silencing of victim-survivors are consistently reported, which ‘add insult to injury’ [65], leading to re-traumatising of victim-survivors [9]. Specifically, participants in these studies experience a kind of stifling or gagging, at many levels, providing strong evidence of ‘network silence’, i.e., actively being silenced [43,66]. In a recent study of 25 academic staff in Irish HEIs, 18 reported experiencing barriers to reporting, and 17 experienced the negative impact of those barriers [52]. Victim-survivors recount having their credibility and interpretation of events questioned [6,9,52,65], dismissed or trivialised [6,65], being gaslit [9], and being persuaded or threatened to maintain silence [13,65]. Thirlwall uses the terms ‘rebuffing’ and ‘rejigging’ to describe recasting the situation in such a way that the victim-survivor feels they have caused the problem or they must take responsibility for addressing it [65].

Victim-survivors report being explicitly positioned as dispensable when weighted against the ‘value’ of an alleged harasser who may be a senior manager, a research star or of strategic importance to the institution [46,61]. In this way complaints are shut down, supressed or sequestered [6,19,65], leading to reluctant acquiescence, self-silencing and a sense of institutional betrayal [9,13,61]. This is the reality of living in ‘the master’s house’.

It is worth noting that a favourable external environment, for example increased cultural sensitivity to gender equality and GBV and support from the government in the form of mandates and guidelines, acts as a facilitating factor for comprehensively addressing GBV in HEs [67]. In this context, Ireland’s recent attention to gender equality in the form of a sector-wide review and action plans, and the National Framework for Ending Sexual Violence and Harassment in Irish Higher Education Institutions, could be argued to act as strong facilitating factors for proactive and transformational policy development. The framework articulates a vision to ensure an institutional campus culture which is safe, respectful and supportive through actions at the level of culture, structures, process and specific initiatives such as active consent programmes and targeted educational activities [68].

Theoretically, this paper draws on institutionalism and, specifically, institutionalised resistance to policies that potentially impact on health and wellbeing. Resistance in an organisational setting is a response to actual or perceived challenges to existing hierarchies of power [69] (p. 394). Agócs argues that when such resistance is embedded in and expressed through organisational structures and enacted systematically by decision makers in organisations in order to preserve and protect their power, it can be characterised as institutionalised resistance [21]. Tauber argues that voicing staff experiences of mistreatment and harassment in academia is seen as a threat to the hierarchy, and organisational actors in this context enact procedures in such a way that victim-survivors are unvoiced and tricked into ‘self-silence’ [22] (p. 5). In this way, complaints act as a lens through which we see the complexities, contradictions and complications of power [19].

Recognising the distinction between the ‘why’ and the ‘how’ [61], and specifically that the non-performance of policy is an exercise of power, the authors aimed to explore staff policies in Irish HEIs, at a time when there is a concerted effort at a national level to address the problem of harassment in the sector, in particular sexual harassment and violence but not excluding bullying and other forms of organisational aggression. We do not ask *if* policies do not perform, as this has been demonstrated previously; see for example [5,9,19,26]. Rather, we ask *how does non-performativity ‘happen’ within stated policy and procedures*?

## 4. Methods

Theoretically, our positionality was of institutionalism and critical organisational theory, with a particular focus on the way organisational processes, such as the response to GBSVH, reproduce wider health and social inequalities through the exercise of visible, hidden and invisible power [70]. As is appropriate to a qualitative study, reflexivity was on-going and iterative, and dependability was evidenced through a transparent research process.

Dignity and Respect, Anti-Bullying and Sexual Harassment policies in HEIs are made available to staff in the form of documents. Documentary analysis was used to systematically examine policy and institutional documents relevant to the research focus. The analysis involved a structured process of document selection, close reading, coding, and thematic interpretation to identify recurring patterns, assumptions, and meanings across the dataset. This typically involves reading, examining and interpreting documents to discover underlying patterns [71,72]. Given the theoretical positioning, documentary analysis was applied through the lens of critical theory, viz., documents were considered as organisational phenomena shaped by the power dynamics in HEIs and with agentic power to maintain those power dynamics. The focus of the enquiry was the interpretation of policy language as a conduit to action and prevention of the known impact on health, rather than a quantitative focus, as prevalence is well established.

At the time of data collection (January–May 2024) there were 12 publicly funded comprehensive universities in Ireland. (The sampling frame included only comprehensive institutions with ‘University’ in their title (n = 12). The non-sampled HEIs in Ireland were six specialised colleges, only one of which was private.) The websites of all universities were searched for anti-bullying, sexual harassment, dignity at work and sexual misconduct policies. Ten had policies publicly available online. The analysis is based on these ten universities.

Once identified, documentation was downloaded and read, extracting information as appropriate to Bowen’s recommendations for documentary analysis [71]. Bias in document selection was minimised by drawing on documents from 90% of the universities and identifying the full population of policy and procedure documents with bullying and harassment or sexual harassment in the title pertaining to university staff. The analysis followed a structured process that involved careful reading, coding and thematic interpretation of each text. Each document was reviewed multiple times to ensure familiarity and to double check interpretation. Documents were analysed through an iterative process of reading and re-reading, during which initial codes were generated and progressively refined into broader analytical themes. This allowed for both descriptive and interpretive insights to be developed from the data.

The analysis was conducted in two stages. The first stage involved an initial round of coding based on qualitative content analysis, using established guidelines and specifications for anti-bullying and harassment policies [73,74,75] (see Table 1) as an analytical framework. This stage focused on identifying the presence, absence, and formal characteristics of key policy components across the document set.

The second stage involved a more interpretive, theoretically informed thematic analysis. Here, documents were re-read and coded inductively to identify patterns and tensions in how anti-bullying and harassment were articulated in practice. This phase was guided by Ahmed’s concept of non-performativity, with analysis focused on examining how non-performativity operates within policy texts, and specifically how commitments to addressing bullying and harassment may be articulated without being substantively realised. Through this process, key themes were developed and refined, forming the basis for the study’s findings.

## 5. Results

The following analysis is based on ten universities, pseudonymised here as Universities B, E, Y, F, H, J, N, P, V, and W (universities were anonymised using letters that did not correspond with any university name or location).

A first reading clarified that the document laid out the purpose and that this pertained to addressing bullying and harassment or sexual harassment and misconduct specifically, and detailed or signposted procedures. Across ten universities, we found 17 documents named as policies that specifically relate to this phenomenon. Several universities had two or more linked documents (e.g., policy statements and procedures in separate documents, staff/student combined or separate) rendering a total sample size of 23 documents. Summary details of how the content of each university’s policy/policy suite aligned with general guidance is provided in Table 1.

Four universities had separate polices for bullying and harassment and sexual misconduct or harassment, and six had separate student and staff policies. Only staff policies were analysed in the universities with separate staff and student policies, given the focus on the workplace as a determinant of health. With one exception, all documents have been updated since the introduction of ESVH in 2019. In their titles, two policies refer to bullying and harassment, eight employed the language of ‘Dignity and Respect Policy’, one employed both sets of terms, three use the terms sexual misconduct, two use sexual violence and harassment, and one uses the term student expected behaviour. None use the term gender/gender-based in their title.

It is noteworthy that the policies are remarkably similar. Without exception all contain opening statements expressing a commitment to creating a respectful environment free of bullying and harassment, with reference to underpinning legislation, for example, the Code of Practice for Employers and Employees on the Prevention and Resolution of Bullying at Work [73] and/or equality law. One also mentioned a moral obligation to recognise the seriousness of bullying, harassment and discrimination. As per good practice guidance, all give definitions of various forms of bullying, harassment and/or misconduct, delineating the scope of coverage, outlining the roles and responsibilities of relevant parties and the steps required for informal and formal processes, and signposting supports and commitment to periodic policy review [73,74,75]. The definition of bullying in each policy is drawn from the Health and Safety Authority Code of Practice [73]. All give examples of behaviours, although the way in which sexual harassment is defined varies. The universities that offer separate documents provide definitions consistent with the National Framework [68] and include both sexual misconduct and harassment. They are considerably more detailed, with two including definitions of sexual violence and one defining coercive control. In the four universities with combined policies, the definitions of sexual harassment are drawn from equality legislation, adapted to sexual harassment and with specific behavioural examples added. They do not reference the National Framework. In all 10 universities, informal and formal procedures are outlined, as are appeals processes, commitments to confidentiality and natural justice, references to malicious or vexatious complaints and the responsibilities of all parties. Only half of the universities refer to monitoring procedures and consulted trade unions or representative bodies in the devising of policy.

As such, the content analysis reveals a strong commitment to good practice and links with appropriate legislation. However, the critically driven thematic analysis yielded findings that expose non-performativity.

Almost all references to gender in the documents are in the context of listing the grounds for harassment under equality legislation, or the commitment to gender balance on investigation panels. Only one document contains a brief reference to ‘respecting’ intersectionality, indicating a clear de-gendering of policies [24].

The policy ‘environment’ is not easy to navigate. There is usually more than one document, in some cases up to five interconnected documents, with embedded links to other functions with the university. Reporting parties are, in effect, following breadcrumbs through a forest. Furthermore, below the surface level of the documents lie nuances that allow non-performativity to ‘happen’, vis à vis the tone and language used and the ‘institutional mechanics’ [19] of the informal and formal processes. These are explored in terms of the emphasis and the language employed in the policies, the ‘happy talk’ of the informal approach and the ‘not-so-happy talk’ of the formal process, all of which are potentially intensified for those with minoritized status.

### 5.1. Tone and Language

The opening statements are, at a superficial level, positive. All institutions claim to be committed to an environment where bullying, harassment and sexual harassment are not tolerated in any form. As can be seen from the examples below, the opening comments are all rather grand assertions containing phrases such as ‘*right to study and/or work in an environment which is free from all forms of discrimination*’ (Y), and a recognition ‘*that sexual misconduct is a serious issue*’ (N). In some cases, a focus on behaviours is explicit within the opening statement, presaging the individualised, atomised, interpersonal misbehaviour approach, rather than an expression of the wider power inequalities in the institution. For example,

*This policy aims to promote respect, dignity, safety and equality in the workplace. Every member of staff should be aware that all forms of bullying, harassment, and sexual harassment are unacceptable, and every member of staff has a duty to behave in an acceptable and respectful manner*

(B and E)

*Workplace bullying, harassment and sexual harassment constitute real threats to the safety, health and welfare of people in the workplace. (University J) is committed to providing a workplace in which bullying, harassment and sexual harassment are not tolerated and in which the dignity of all staff is protected…*

(J)

There is reference to the wider institutional environment, but this is limited to opening statements and tends to disappear as the policies delineate processes and procedures. Policy statements are peppered with words such as ‘respectful’, ‘equality’, ‘inclusivity’, ‘rights’, ‘safety’, and ‘zero-tolerance’, conveying an optimistic belief that a policy, well intended, will potentially eliminate problems such as sexual harassment [76]. This is essentially ‘happy talk’ [77], an attempt to convey a pleasing, overly simplistic narrative and statement of good intent, that conceals complexity, in particular power differentials. Yet simply stating the importance of a ‘*safe, supportive, consenting, compassionate, positive and respectful campus*’ (B) does not mean that there is one [19], but rather reflects an uncritical assumption that asserting the wrongness of sexual harassment is sufficient to prevent it [78]. Furthermore, the tone is unemotional, intending to convey rationality and impartiality, a bureaucratic stance that is essentially patriarchal and serves to disguise power relations that underpin abuse and violence in university workplaces [79].

Three policies explicitly encourage staff to come forward to seek support and assistance, bearing in mind that ‘encouragement to come forward’ is ethically questionable in a hostile environment [24]. For example,

*You have the right to disclose experiences of unacceptable behaviour while studying or working, to be listened to, to seek support and to have the issue resolved. We encourage you to come forward to seek support and assistance in resolving any issues of bullying or harassment, and to explore informal and formal options available for resolution. You can be assured that we will act sensitively to all cases of bullying and harassment*

(Y)

Conversely, of equal concern is how some policies border on discouragement, not to protect the victim-survivor, but as a way of protecting the institution. This can be seen in the form of doubt-raisers, for example self-audit checklists to be filled out before enacting procedures, implicitly questioning victim-survivors’ understanding of their experience. The tone can appear scolding, and there are subtle warnings planted throughout the documents, including reminders of the seriousness of proceeding to investigation, or the failure to maintain confidentiality. For example,

*If an individual thinks that they have been bullied, they need to seriously consider whether this is the case before making a complaint*

(B)

*‥that any persons (i.e., complainant, respondent, witness(s), investigator(s), other staff involved) found to be in breach of confidentiality relating to a Formal Investigation will be dealt with through the Disciplinary Procedures*

(V)

Although ‘false allegations’ are in fact very low in prevalence [24], nine of the ten universities issue unambiguous warnings about vexatious or malicious complaints;

*(The) University will treat seriously any such allegations which are deemed to be malicious and without foundation. The person responsible for such allegations, or any parties involved in the support of such allegations, may also be the subject of disciplinary action*

(N)

Vexatious complaints do occur and bullies do in fact weaponise and abuse policy, and the critique offered here does not lose sight of this. However, placing of these warnings, in some cases in the first few pages of a lengthy document, is telling, as is the fact that in most documents this is the only place where the possibility of disciplinary action is explicitly referred to. While malicious complaints are a reality in institutions, if an individual is making a complaint with malice aforethought, they are unlikely to check the policy first to see if they are allowed to. Bearing in mind that genuine victim-survivors of bullying and harassment are most likely to be uncertain, experiencing shame and embarrassment [80], such ‘warnings’ placed so early are more likely to have the effect of adding to their self-doubt and apprehension. Many of the policies do not define what vexatious or malicious actually means and thus run the risk that the victim-survivor can interpret vexatious as ‘difficulty with proof’, already a challenge in a system biassed against women and racial minorities [24]. Only two universities actually outline that a vexatious or frivolous complaint is one that ‘*is made with the knowledge that it was untrue*’ (P, Y) but one contains the wholly unreasonable caveat that vexatious complaints are also ‘*where one person maliciously complains of someone allegedly engaged in sexual misconduct with a third party, without fully exploring the veracity of the claim*’ (P). Strong warnings follow about the consequences for the ‘wrongly’ accused which could also have the effect of trivialising the ‘disruption’ sexual misconduct has had on the victim-survivor:

*A malicious complaint has the power to disrupt another person’s life to a significant extent and the potential damage should not be underestimated. Being accused of sexual misconduct can have a serious impact on any person and reduce their reputation in the eyes of others, even if later shown to not have been proven. Those making complaints—and those involved in early assessment of the circumstances of a complaint—should always be mindful of the context and situational aspects of the event and accept the different perspectives and points of view different people bring to the same event. Making a malicious complaint, if proven, can have serious implications for the employment/studies of the person making such a complaint and this includes disciplinary action, where established*

(P)

Equally unjust is the designation of ‘*a refusal to accept a reasonable decision arising from the application of the Sexual Violence and Harassment (SVH) Procedure*’ as vexatious, in University F.

Other ‘warnings’ include signalling to the victim-survivor that they could (but should not) mix up ‘normal’ disputes and disagreements and those that can be classified as bullying; for example,

*Interactions between individuals may become emotional, heated or evoke strong feelings however this does not necessarily mean that the interaction fits the definitions of bullying, harassment or sexual harassment as outlined*

(J)

Statements such as these de-contextualise the experiences of the reporting party and imply a false simplicity to what are complex, circular, degenerated social relationships, undergirded by gendered, racist and ableist power abuses. They are a form of victim-blaming (‘you have mis-read the situation’) and could potentially have the effect of undermining the reporting party’s self-confidence and willingness to proceed with a report. While substantial effort has gone into detailing the implications of vexatious complaints, comparatively less effort is visible with regard to post-investigation outcomes for those found to have engaged in bullying and gender-based harassment or for the complainant.

Interestingly, four of the policies presented elements of their documents in the second person, making for a more user-friendly document:

*You can be assured that we will act sensitively, and disclosures will be carefully and thoughtfully addressed through a process that is transparent and clearly communicated to all individuals involved*

(B)

However, the third person was more typical, thus placing the ‘narrator’, the university, outside the narrative and creating emotional distance; for example,

*Those who feel that they are currently being, or have been, bullied, harassed or sexually harassed or otherwise treated in breach of the policy, should use this policy and procedures for their protection (…) All employees and non-employees will be expected to comply with this policy and the University will take appropriate measures to ensure that, insofar as practicable, workplace bullying, harassment and sexual harassment do not occur*

(J)

This more formal style is presumably used to emphasise objectivity and can also be read as a degree of separation and/or a defensiveness in tone, protecting the institution rather than the reporting party. The tone is overtly authoritarian in a number of policies. The documents written in this manner can be seen as authorless and agent-less, an exclusionary discourse that obscures institutional responsibility [76,79].

The terminology for the reporting and responding parties sends subtle signals. Only one university comprehensively employs the terms reporting party and responding party, while in three other universities both reporting party and complainant are employed. In six universities the term employed throughout is ’complainant’. In using the word complainant, a legalistic tone is introduced, evidence of ‘criminal justice drift’, the adversarial and procedural turn influenced by the criminal justice system [81]. Ahmed’s comment that ‘*words carry a charge*’ resonates. ‘*Words are important, they can make you feel that you are the problem, that they point to you, but not to the perpetrator or the institution*’ [19] (p. 16). Arguably the reporting party is wrong-footed from the start, potentially cast as a troublemaker, someone that no-one wants to listen to. The responding party, on the other hand, is usually called the respondent or, in the case of three policies, the ‘person’ against whom a complaint is made. This contradicts the claims to impartiality and fairness found in the opening statements of many policies and is consistent with analyses of university responses to sexual violence in UK universities; see for example [24,81].

### 5.2. More ‘Happy Talk’—The Informal Approach

Sixteen policies outlined informal procedures as a first step for ‘*dealing with any such issues should they arise*’ (J). This is presented as a way to ensure *‘the minimum of conflict and stress for the individuals involved*‘ (H). How the informal process is expected to take place is illuminating in respect of non-performativity, and very unlikely to protect individuals from stress. It is naïve, unrealistic and potentially offensive. It individualises the issue. It implies that complaints are aberrant and exceptional, rather than regular, even routine abuses of power.

The reporting party is encouraged, in some cases ‘strongly’ encouraged, to approach the alleged perpetrator directly. This practice is supported by claims that this is ‘often’ the best way to resolve matters, on the grounds that the behaviour may not be intended or ‘*the person may be unaware that their behaviour is causing distress*’ (H). For example, University N confidently claims that ‘*…once the person who feels they have been bullied or harassed has made the person(s) engaging in the unwanted behaviour aware of its effect, it is frequently the case that the latter desists and appropriate relations are restored between the parties*’ (N).

Such statements betoken more ‘happy talk’ and are not supported by evidence. The lived experience of victim-survivors strongly indicates that, from victim-survivors’ perspectives, perpetrators are very aware of the distress they are causing and are acting with intent, bias in self-selection notwithstanding. Further, victim-survivors of GBVH fear that letting the perpetrator know that their behaviour is offensive will lead to more harassment [76] and retaliation for raising the issue. Ahmed comments on this development caustically: ‘*a bully with a complaints procedure is a bully with another weapon*’ [19]. In the context of GBVH as an abuse of power, perhaps power along several bases (e.g., position, social, expertise), this is not an unrealistic assessment. It is well established that for women and those of minoritized status, the risks of approaching the harasser directly are much greater [32,82].

It is allowed in most policies that the reporting party may not wish to confront the perpetrator and may ask another person to do so on their behalf. This is also unrealistic, as it potentially places another person at risk. Moreover, many policies use words that imply deficit or weakness in some way on the part of the reporting party in these instances; being *uncomfortable or unable to have the conversation directly with the alleged perpetrator* (H, J), or who may find it *too difficult or embarrassing* (V). Not wishing to discuss the alleged perpetrator’s actions directly with them is more likely to be based on a very realistic awareness of the risk involved, and therefore a self-protective strategy.

The hinting at deficits on the part of the victim-survivor not only pathologises them, but reinforces incident-focused assumptions, masking power abuse and sidestepping the social and organisational conditions that make such behaviours possible [78]. It is a re-packaged version of ‘just say no’, a de-contextualised framing of bullying and sexual harassment as an individualistic paradigm, as something that occurs between unfortunate and misguided individuals. As such, the victim-survivor is expected to bear the responsibility for stopping it, conveniently absolving the institution of the responsibility for challenging the underlying exercise of power, perpetuation and discrimination [24,76,78,79]. Although two policies allow that safety is an issue, they still propose an attempted resolution through a direct conversation. One university actually recommends the following: ‘*if you do not want to face the person, … put your concerns in writing directly to the person concerned. Explain what it is about the behaviour that is upsetting you and ask for it to stop*’ (P). Victim-survivors understand how their experiences are subtle or not-so-subtle abuses of power, and cannot nor should not be asked to confront the person who is abusing their power. To suggest that they do is a wilful disregard of power relations and the attendant vulnerabilities of women and those of minoritized status in HEIs. In cases of sexual misconduct, it is completely inappropriate and a risk to physical and psychological safety to expect victim-survivors to explain that risk to the perpetrator. To place concerns directly in writing increases the risk to the victim-survivor especially in climates of criminal justice drift [81] where to name behaviours opens them up to risk of accusations of making allegations or ‘ringing bells that cannot be unrung.’ This contributes to poor outcomes for reporting parties [25,26], who experience what Bull describes as institutional listening while silencing [26].

Another example of ‘happy talk’ can be found in the confidentiality clauses, also noted by (Ahmed [19]). All policies contain clauses about confidentiality, with regard to mediation, formal processes, witness interviews and the outcome of investigations; for example, ‘*All complaints will be dealt with promptly and in a confidential manner in accordance with the agreed procedures and with due respect for the rights of the parties involved*’ (F).

Claims to confidentiality are reassuring at the surface level but are unworkable and unlikely to be trusted. It is hard to see how it can realistically occur, where people may be moved (precautionary measures) and several staff are involved as screening panel members, witnesses or on the investigatory team. Further, confidentiality clauses create atomisation, where treating each complaint separately allows serial abuses of power to go unrecognised [19]. More cynically, Ahmed claims, this also functions to keep those who complain apart from one another [19] and has the additional effect of employees generally not knowing whether complaints, if they are upheld, are sanctioned or what those sanctions are. Any assessment of the organisation’s record in dealing with misconduct is fully concealed by these confidentiality clauses, and, as such, they are fundamentally unaccountable. In this context, low trust is unsurprising.

### 5.3. The Formal Process/Not-So-Happy-Talk

A ‘formal’ process is outlined in every document, a process that we argue is inherently unpredictable and essentially defensive practice on the part of the institution. Across all policies, with minor variations, this process entails a submission of a formal complaint including ‘precise details of alleged incidents of bullying including their dates and names of witnesses, where possible’ (B), a screening of the complaint and a decision based on this screening whether to proceed to investigation, usually described in terms of whether the behaviour in question ‘falls within the definition of bullying, harassment and/or sexual misconduct and the scope as outlined in Bullying and Harassment policy and Sexual Misconduct policy’ (Y). This ignores the impact of trauma on memory. However, even where memory is not impacted by trauma, the requirement is likely to be interpreted as whether the reporting party is ‘correct’ about the classification of behaviours and whether it is sufficiently serious. Although these are arguably legitimate considerations from the employer’s perspective, they act as doubt-raisers to a person who is likely to be already on shaky ground in terms of self-confidence as a result of what they have experienced, and reinforce the unpredictability of the process. Some policies impose a timeframe from the ‘alleged incident’ regardless of how realistic it is for traumatised persons to come forward with a report rapidly. No information is given about what happens if the ‘alleged incidents’ of GBVH reported do not fall within the policy, exposing an unrealistic binary position. In seven of the ten universities, both reporting and responding parties have access to the statements of the other and of witnesses. In three, the responding party has access to the reporting parties statement but not conversely. In one university the respondent can view the reporting party’s statement before the screening process, which leaves the reporting party entirely exposed.

There is evidence of criminal justice drift [81]. Documents are peppered with terms that reinforce the quasi-legal approach [25] taken by the universities; for example, there are interviews, witnesses, and decisions that are made on an ‘evidentiary basis’ or on the ‘balance of probabilities’, supported by the ‘burden of proof’, all of which speak to an institutional defensiveness. This approach has been criticised in the literature as overly procedural and adversarial and to risk the re-traumatising of victim-survivors [81].

In the documents we examined, the screening person/panel decides if an investigation is warranted and if it is, investigators are appointed, usually including at least one external expert, although policies typically do not detail what qualifies as expertise in this context. Terms of reference and timelines are agreed; a series of interviews with the reporting and responding parties and witnesses and exchanges of reports then take place, before the investigators arrive at a decision. In essence, the claim to objectivity and neutrality means the reporting party is being investigated as well as the respondent.

One university applies the wholly unrealistic condition that parties must agree to ‘*…the prohibition on making any contact, directly or indirectly, with the respondent or any potential witness on any matter related to the complaint, and that any breach may result in disciplinary action*’ (V). Reporting parties are in effect experiencing ‘institutional listening while silencing’ [26] and are indirectly precluded from support seeking. To prohibit any contact on matters related to the complaint with the respondent or potential witness is totally unworkable, given that GBVH ‘behaviours’ are embedded in everyday interactions, and indeed everyday situations can be used by harassers as bullying tactics. A reporting party could jeopardise their career by avoiding contact or give grounds for disciplinary action. Further, if the ‘respondent’ is alleged to have committed assault, one might ask why the priority is not to protect the victim-survivor, rather than instruct them to ensure no contact takes place. These provisions add to the accumulating weight of presumption and exposure for the victim-survivor.

The decision made by investigators is usually ‘complaint upheld’, ‘not upheld’ or ‘vexatious report’. Most policies explain that ‘compliant not upheld’ is not the same as a vexatious complaint, although one university elides the two outcomes in a way that has a distinctly threatening overtone. For example,

*If a complaint is not substantiated, that is, if it is concluded that on the balance of probability bullying, harassment and/or sexual harassment has not occurred, the matter will be considered to be closed by the Senior HR Manager … unless there is evidence to support a view that the complaint was malicious or vexatious. In such circumstances, the Disciplinary Procedures will be invoked against the complainant by the Senior HR Manager on the relevant campus*

(V)

All documents lay out the formal process with, unsurprisingly, a high degree of formality. While in a few cases the text is intended to be as user-friendly as possible, in most policies it is highly procedural, with multiple conditional steps, clearly mimicking criminal justice procedures. For example, five policies detail up to 11 steps, and one has seven pages explaining the formal process, principles and requirements.

In universities where there are two separate policies for bullying and sexual harassment [6] the reporting party may find that their situation does not fall under one policy but possibly the other, in which case they must ‘return to go’ and start over without knowing if the (separate) screening panel of the second policy will agree that it falls under this policy. It is hard to imagine a less victim-centred procedure.

In three of these universities the sexual misconduct policy signals that if the *Garda Síochána* (Irish Police service) are involved the institutional process is potentially paused until the state has completed its investigation: ‘*If An Garda Síochána are managing a formal process, the University might pause/adjourn its internal case, pending the conclusion of the criminal process. A decision to pause/adjourn will be made by the preliminary evaluation meeting group informed by An Garda Síochána and legal advice.*’ Given that legal or criminal processes are typically lengthy, the victim-survivor is potentially at on-going physical and psychological risk if the university pauses all action, and is potentially re-traumatised.

All universities include appeals processes. Appeals are on procedure, not outcome. A procedural appeal is on the procedure as laid out by the institution, not whether the procedure itself is fair or just or allows for injustices in the system. Two policies explicitly refer to the inclusion of an external independent person on an appeals panel, two include members of the investigatory panel on the appeals panel, and one refers the appeal internally, to a senior member of management. All others are vague about the constitution of the appeals panel, for example referring to ‘appropriate persons’ and to membership being at the discretion of the Director of HR or the President.

The formal process is quintessentially bureaucratic, stemming from an ideology of rationality, in which procedural, logical, linear practices are privileged, and are placed in binary opposition to emotionality [83]. This has the effect of creating an illusion of neutrality and detachment [76,84], made explicit in the frequent references to ‘natural justice’ and reinforcing gendered, racialised and other intersectional inequalities [12,24]. Seven of the ten universities pronounce their adherence to the principles of natural justice; for example,

*All parties involved in an investigation will be afforded natural justice and will be treated with fairness and sensitivity and with respect to the need for confidentiality with all parties concerned*

(F)

One university reinforces this with a statement about the presumption of innocence:

*The aim of the University shall be to balance the principles of natural justice, the health and safety of staff and students, the integrity of any possible criminal investigation, and the right to the good name and presumption of innocence of all parties concerned*

(B)

Reporting parties may interpret this claim to fairness, and especially the presumption of innocence, with a high level of cynicism in the context of their experience of bullying or harassment from people who have more power in the institution and who have used that power to mistreat or harm them. They may expect a ‘reckoning up’, as evidenced in Phipps’s analysis of how power relations in HEIs facilitate the protection of perpetrators in cases of sexual harassment who are ‘institutional breadwinners’ [61], ‘research stars’ or senior academics [46]. Bullying and harassment are both gendered and racialised and those who have been exposed to it in this way are already aware of the stark injustice inherent in their experience. Natural justice chest-beating is far from reassuring when those with minoritized status across the institution have already experienced injustice on an on-going basis. Natural justice assumes a level playing field, when in reality the HEI playing field is far from level in respect of including risk of exposure to bullying and harassment, especially sexual harassment and violence.

## 6. Discussion

Public health operates at the level of the state, the community, its institutions and its organisations. Violence also operates at these levels and is constitutive of public ill health. HEIs are part of a system of wider and interacting determinants of public health, and are embedded in this process, in terms of the enactment of violence and the potential prevention and amelioration of it. Universities, as microcosms of society, reflect wider inequities and power relations that impact on health, but through the non-performativity of their bullying and harassment policies, preserve these power relations, rendering this an issue of significant importance. We argue that HEIs need to look inside the ‘Master’s House,’ and critically assess and even dismantle their ‘tools’ for the prevention and management of bullying and harassment in the interests of public health. We also advocate that such policies should be cognisant of the national public health policy that explicitly states that health is created and lived by people where they learn, work, play and love [85]. It appears rare that public health policy intersects, if at all, with the development in universities of workplace policies on violence or harassment particularly when they have a bearing on employee health. This is worthy of further consideration particularly as part of the broader focus of the International Health Promoting Campus Network (IHPCN) as their work is grounded in the 2015 Okanagan Charter, an international charter for health promoting universities and colleges. In particular, this charter cites that health promoting universities and colleges transform the health and sustainability of our current and future societies, strengthen communities and contribute to the wellbeing of people, places and the planet [86] (p. 2). The guidance of the charter advocates that ‘health promoting universities and colleges infuse health into everyday operations, business practices and academic mandates. By doing so, health promoting universities and colleges enhance the success of our institutions; create campus cultures of compassion, well-being, equity and social justice; improve the health of the people who live, learn, work, play and love on our campuses; and strengthen the ecological, social and economic sustainability of our communities and wider society’ [86]. The charter calls for the embedding of health into all aspects of campus culture, across the administration, operations and academic mandates, and this should include ethical leadership, policy formulation and implementation.

In this article we have analysed the bullying and harassment/sexual misconduct policies in ten Irish higher education institutions, seeking to identify how non-performativity, as observed in Ahmed’s analysis of harassment complaint experiences, happens, using the theoretical lens of institutionalised resistance.

When power is challenged in organisations and the status quo is perceived to be threatened, institutionalised, system-supporting resistance occurs [21,87]. Institutionalised resistance is an exercise of ‘second face of power’, operating through the framing of problems and their solutions and controlling the parameters of various policy actions, in order to benefit certain groups, i.e., those with power and authority in the institution, at the expense of others, i.e., those with less or limited power [88,89]. Making a complaint is a politicised action [19], since it is inherently a challenge to power, and more powerful actors in the institution have a vested interest in resisting such challenges and thereby maintaining their position [59]. As Ahmed unambiguously states, ‘power is not simply what complaints are about; power shapes what happens when you complain’ [19] (p. 24).

We argue that the non-performativity of GBVH policies in HEIs is an expression of institutionalised resistance, insofar as non-performativity is an exercise of power that serves to protect the institution and the powerful groups within it, at the expense of those with less power relatively, and especially those who are minoritized and marginalised.

Ahmed observes that ‘you can stop something by making it hard to use’ [19] (p. 31), as evidenced by the low levels of reporting of GBVH in HEIs. This effectively protects the institution from exposure and reputational damage. The depersonalised and authoritarian tone of the policies analysed here, the use of the legalistic terms and pejorative terms such as ‘complainant’ and ‘vexatious’, and the subtle and not-so-subtle warnings all potentially deter victim-survivors. They place the victim-survivor in a procedurally disadvantaged and accusatory position from the start, having to account for themselves within a quasi-legal court-like system in which they are being investigated along with the perpetrator. These aspects of GBVH policies, the ‘tools of the Master’, reinforce one another, creating a concentrated effect. The cumulative weight of the questioning, scarifying, and victim-blaming inherent in the procedures cannot but compound the well documented psychological health outcomes of GBVH.

Despite grand opening statements about the commitment to fairness and equity, policies to prevent and address GBVH in Irish HEIs raise significant concerns about procedural fairness and appear to embed mechanisms that serve to reinforce, preserve and privilege systemic control. The framing of the problem protects institutional power. Consistent with the findings of Bull and Page [25], we find that GBVH is presented as a problem of individual irregular behaviours enacted by an identifiable perpetrator on an identifiable victim that can be addressed through this identification and ‘resolved’ by intervention for one or both individuals. This is especially evident in the sections of policies dealing with an informal approach, which are characterised by a ‘Disneyfied’ [90] narrative, using phrases and descriptions that suggest minor conflicts or unfortunate misunderstandings, a patronising simplification [78] and de-contextualisation of what is a complex, multi-faceted organisational problem [2]. The errant behaviours are downplayed, to be resolved by a chat. The various provisions against retaliation and victimisation are also an individualistic framing, as are the exhortations for good behaviour found in opening statements. This framing serves to deflect attention from the social and structural inequities that pervade academic institutions, how these inequities surface as violence and how individual and social and structural relations underpinned by inequities are interrelated, mutually constitutive and sustaining [34].

The lived experiences of the victim-survivors of GBVH demonstrate an experience beyond an ‘incident’ or a set of discrete incidents. GBVH is experienced as subtle and devious, involving sophisticated strategies on the part of the perpetrator, often disguised in the context of HEIs as career advice or ‘robust’ feedback. Being bullied or harassed for staff is an evolving process, unfolding against a backdrop of legitimate organisational processes and interacting with those processes. It does not necessarily proceed in a straight line, and indeed can be circular and ‘messy’ [19], with deliberate intent, including the desire to punish the victim or sabotage their career. Oversimplified representations make more complex representations less possible or likely [78] acting as a barrier to making a complaint [27] and maintaining the existing status quo.

Relatedly, this framing of the ‘problem’ of GBVH that obscures the power plays and abuses that underpin it is the way in which the processes assume an honesty and integrity on the part of the ‘respondent’ insofar as the various steps are laid out with no provision for the perpetrator that refuses to cooperate. There is no evidence in any policy of recognition of DARVO (Deny, Attack, Reverse Victim and Offender roles), where the alleged perpetrator deflects blame and responsibility when confronted for their abusive behaviour, assumes a victimised position and declares the victim to be the true perpetrator, a phenomenon well documented in the bullying and harassment literature and the sexual harassment literature [12,91,92,93]. The naivety at best or wilful blindness at worst of this approach contrasts with the experience of victim-survivors, and also protects the institution from the reality of accepting much GBVH as upsurges in the continuum of symbolic violence always bubbling along in HEIs as a result of structurally created power differentials [24,34].

Confidentiality clauses also serve the interests of the institution since they prevent serial GBVH from being detected [19]. Confidentiality clauses are ostensibly driven by the considerations of ‘unfairness’ to alleged perpetrators, consistent with the observation that institutionalised resistance co-exists with declarations of commitments to equality and to ending discrimination when it actually acts conversely [21,87]. Confidentiality clauses are exercises of stealth power in effect, ensuring that abuse can continue unabated; they give space to perpetrators to spin and reframe complaints as personality issues and protect them from having to acknowledge and ‘own’/be accountable for behaviours. They facilitate the criminal justice drift that appears to give more rights to responding than to reporting parties [26,81] and thereby legitimise and reinforce a culture of not addressing power abuses. Sealing off ‘cases’ from other investigations is an act of resistance to challenges to exploitative power relations.

The formal process, which victim-survivors enter if the informal process fails to address the situation, is also a subtle exercise in stealth power. In contrast to the informal process, it is rigid, highly procedural and fundamentally adversarial, mimicking the criminal justice system [81]. It puts the victim-survivor ‘on the stand’ in a way that does not happen with other types of crime/transgression. This is problematic for a number of reasons, chief among them the re-traumatising of victim-survivors and the divorcing of incidents of ‘deviant’ behaviour from broader structures of gender inequality [81]. While a punitive criminal justice approach can be appropriate for some victim-survivors, it is not the only one. Studies of sexual violence victim-survivors’ perceptions of justice reveal a spectrum of less adversarial justice mechanisms, for example recognition of harm inflicted, educational initiatives, ensuring future protections in the institution; essentially restorative and transformative justice processes [81,94]. These justice mechanisms, however, potentially challenge power structures. Punitive justice responses on the other hand absolve the institution from having to address the conditions which create the problems of abuse in the first instance, and strict adherence to them, to the exclusion of other forms of justice, is, we argue, a way of maintaining the status quo and a form of institutional resistance.

Critically, the centrepiece of policies is the ‘determination’ issue: the determination as to whether the situation does or does not come under the policy, and, if it does, whether the complaint is upheld or not. There are in fact multiple determinations as the victim-survivor moves through the informal and formal processes, and each time the reporting party is assessed for credibility according to an undefined and/or an unmeetable standard. The screening panel, which decides on whether the reported behaviour ‘falls under the policy or not’ comprises senior staff, typically the Director of HR, the Registrar or a Vice-President. As such, the power to decide if a formal complaint is to be allowed rests almost entirely with senior management in the institution and in this way the institution is exercising power to protect power [21]. While the investigatory panel usually includes external members, they are selected by senior members of the institution, and if an appeal is raised, this process is almost wholly returned to internal senior staff, neatly stitching the seam of power and control.

‘Determination’ is an expression of institutionalised resistance, a ‘tool’ chosen by the Master to protect the Master’s house. Determination is evidence of criminal justice drift, borrowing from a legal process which is driven by actual law, rather than perceptions and subtle interpretations of complex social and organisational relations. It ignores the fact that binary logic is an inappropriate framework for GBVH and perception is crucial, not a confounding variable to be erased. It emphasises the institution as a legal entity and reinforces an individualistic framing and a case-management approach to handling complaints [2]. It focuses on who did what to whom, when and where [78], and whether certain words were used or certain acts took place [19]. While these details matter to the reporting party, the narrow focus on them within the formal process allows them to ’become a substitute for a conversation about the conditions out of which they have emerged’ [78] (p. 131). It is adversarial, clearly leading to a decision in which one party wins and one party loses, the latter usually the party with minority status, rendering the process a risky one for those who read the power dynamics correctly. Finally, but perhaps fundamentally, the coda to institutionalised resistance is how the policies and procedures to address GBVH are power washed, concealing the real exercises of institutional power, and it itself is an exercise of stealth power.

Despite the obvious power dynamics inherent in both an informal process that expects victim-survivors to confront or explain the harm they experience to perpetrators and the legalistic determination process, it is notable that there is very little reference to power in the various documents. The policies are implausibly blind to the risk posed by these recommendations for an informal approach, especially in the case of sexual misconduct. Also notable is the absence of reference to gender, beyond listing it as grounds under equality legislation, or in one case, ‘respecting’ intersectionality. This is a clear example of a shearing-off of gender, or de-gendering; in essence, it is power washing [12,24]. The limited use of the word violence also serves to disguise power [24], and in fact the tone of policy documents is one of striving for neutrality as evidenced by the legalistic, authoritative tone implying a binary logic [83]. This creates a false neutrality that conceals bias [76], well established in the context of higher levels of perpetration of GBVH (especially sexual) by men (who hold the majority of senior positions) and higher levels of exposure for women and by minority groups [5,6,42]. The claims to ‘natural justice’ ignore that the gender regime in HEIs is inherently unjust, a far from level playing pitch for women and those with intersecting minority status [12]. This ‘power washing’, a particularly subtle exercise of second face power, clearly serves the interests of the powerful within the organisation.

## 7. Limitations

The limitations of this study include the focus on one legal and policy jurisdiction and the relatively small sampling frame (i.e., only 12 public universities at the time of data collection).

## 8. Conclusions

However, despite these limitations, we conclude that this documentary analysis of 23 policy documents offers important insights into this environmental tool that impacts on employee health. Despite containing many of the elements of ‘good practice’ [74,95], GBVH policies appear to facilitate non-performativity through the tone of the documents, the language employed and the way in which the informal and formal process are prescribed, and in so doing they preserve institutional power and the status quo. GBVH is framed as an individualistic, incident-based problem, to be resolved case-by-case, ensuring that the ‘determination’ processes, ersatz-legal procedures, are undertaken by principally internal senior staff. Confidentiality clauses prevent the uncovering of serial misconduct, and how power abuse associated with structural inequity is the root of the problem. Warnings about vexatious complaints serve to unnerve reporting parties and suggest that they may be the trouble maker. We find policies to be de-gendered, de-politicised and power-washed, cleverly avoiding reference to power or the reality of its exercise. In this context, it is interesting to consider who the real audience for GBVH policies is—victim-survivors or governmental and institutional review processes.

Meaningful reform will require HEIs to employ alternative tools capable of unsettling these entrenched institutional arrangements, and this will require strong, ethical leadership. We suggest new tools in the form of victim-centred, trauma-informed, remediation- and restorative-based approaches that are based upon the key principles of health promotion from the Ottawa Charter [16] and the Okanagan Charter [86] where the settings approach is comprehensive and empowerment is core. Nothing short of transformative leadership will address this problem. The issue is not one of refining existing tools; rather, new tools are required, alongside a fundamental transformation of institutional structures. The policy documents that uphold and preserve inequity were created by the architects of the inequity—the Master, building his house.

As a potential way forward, we recommend that HEIs *move beyond legalistic and bureaucratic understandings of bullying and harassment*. We recommend focusing on the problem as a public health, health promotion and occupational health issue that is settings specific, which has to be explored as a way of helping people to work together, rather than an adversarial process from which one party will emerge as a ‘loser’. The recommendations of Frasca [96] in respect of remediation-based, restorative justice approaches which focus on who has been harmed, what their needs are and how they can be addressed merit serious consideration, and we recommend future research studies on implementation and evaluation.

We recommend recognising the harm both from the experience of GBVH and the punitive institutional response, and that leaders respond with integrity and openness. Future research studies could focus on consultation with those who have experienced GBVH about contexts in which the policy can come into use, the contingent and unexpected outcomes (e.g., DARVO), and explore potential innovative and creative approaches [76]. The ‘problem’ of GBVH cannot be addressed independent of the continuum of gender and racialised power dynamics that pervade higher educational institutions.

We note that these phenomena are inevitably connected by the common denominator of power abuse and hold each other in place as a circular mutually reinforcing cause and effect relationship [37]. This interdependence not only entrenches existing hierarchies but also makes interventions difficult, as addressing one issue in isolation risks leaving the underlying structures of abuse intact. In effect this normalises abuse and obfuscates accountability. This is incumbent on HEIs, given their key role in determining public health.

## Figures and Tables

**Table 1:** Content analysis based on guidelines for good practice in devising anti-bullying and harassment policies.

Category	Item	Number of Universities with Evidence of Item
**Title and Date**	The policy is clearly titled and dated	10 universities
**Aim**	Has a stated aim or purpose Employs simple, direct, unambiguous language, e.g., opens with an unambiguous statement of intent re opposition to bullying/sexual harassment	9 universities
**Consultation**	Created in consultation with trade unions and/or employee representatives, Health and Safety Authority, the academic literature	5 had consultations
**Underpinning Legislation**	Makes reference to underpinning legislation	10 identified health and safety and/or equality legislation
**Ownership**	Is ‘owned’ by responsible person (e.g., signed or person with responsibility for the policy named)	Stated or implicit in 10 universities
**Values and Principles;** **Natural justice** **and Respect**	Commitment to confidentiality Commitment to principle of natural justice to those alleged and to fair and sensitive treatment Reference to all parties treated with sensitivity and respect	10 gave confidentiality, natural justice and all party respect commitment
**Scope**	One policy for staff and students or two separate policies	6 had separate policies
	One policy for bullying including sexual harassment or two separate policies	4 had separate policies
	Contains a declaration of non-tolerance of bullying by multiple parties, e.g., employees, clients, customers or sub-contractors, and also at various locations if university-related event(s)	10 included multiple parties
**Definition**	Describes/defines what is meant by bullying and/or sexual harassment, including a non- exhaustive list of examples Contains a declaration of the commitment to the protection of complainants from victimisation	10 provide detailed definitions 6 commit to protection from victimisation
**Process**	Clear steps for process, including informal, mediation and formal. Process/procedures should be clear and easy to follow, including flowcharts, forms or templates that facilitate staff and prompt key information Signposts ‘designated contact persons’ Balance of attention (informal vs formal) Commits to timeliness, giving timeframes Information on who conducts investigations, e.g., internal, external List the possible outcomes for complaints Contain an appeals process for both parties Identifies possibility of malicious complaint and sanction for same	10 outline clear steps for process and signpost designated persons 9 are balanced with regard to informal vs formal 10 commit to timeliness 7 give information on persons investigating 6 list all possible outcomes for complaints 10 outline an appeals process 10 identify possibility of malicious complaint and sanction
**Support**	Signposts supports for parties	10 signpost supports
**Responsibilities**	Responsibilities outlined for the which/how many parties	9 outline responsibilities of various parties
**Monitoring**	Evidence of commitment to systematic data monitoring and policy review of absence data and exit data; regular engagement and health surveys, which ask about bullying behaviours, training offer, attendance and evaluation data	5 refer to monitoring procedures
**Commitment to prevention**	Acknowledge prevention is best way to avoid bullying and make a commitment to creating an anti-bullying culture	1 acknowledges culture assessment and change is relevant to preventing B/SH

## Data Availability

No new data were created.

## References

[1] Krug E., Dahlberg L., Mercy J. (2002). World Report on Violence and Health.

[2] Bondestam F., Lundqvist M. (2020). Sexual harassment in higher education—A systematic review. Eur. J. High. Educ..

[3] Keashly L., Neuman J., Lester J. (2013). Bullying in Higher Education:What Current Research, Theorizing and Practice tell us. Workplace Bullying in Higher Education.

[4] Lipinsky A., Schredl C., Baumann H., Humbert A., Tanwar J. (2022). Gender-Based Violence and Its Consequences in European Academia, Summary Results from the UniSAFE Survey.

[5] MacNeela P., Dawson K., O’Rourke T., Healy-Cullen S., Burke L., Flack W. (2022). Report on the National Survey of Staff Experiences of Sexual Violence and Harassment in Irish HEIs.

[6] Naezer M., Benschop Y., van den Brink M.C.L. (2019). Harassment in Dutch Academics. Exploring Manifestations, Facilitating Factors, Effects and Solutions.

[7] Mazzone A., Jones E., Freeney Y., O’Higgins Norman J. (2022). Report on the National Survey of Staff Experiences of Bullying in Irish Higher Education Institutions.

[8] Walker J. (2025). Trauma, Power, and Psychological Safety: Understanding the Mental Health Impact of Workplace Bullying. Healthcare.

[9] Hodgins M., Fahie D., MacCurtain S., Kane R., McNamara P.M. (2024). “My Core Is Cracked”—Bullying in Higher Education as a Traumatic Process. Int. J. Environ. Res. Public Health.

[10] Kivimäki M., Virtanen M., Vartia M., Elovainio M., Vahtera J., Keltikangas-Järvinen L. (2003). Workplace bullying and the risk of cardiovascular disease and depression. Occup. Environ. Med..

[11] Boddy C.R., Boulter L. (2025). HRM’s response to Workplace Bullying: Complacent, Complicit and Compounding. J. Bus. Ethics.

[12] Bull A., Shannon E. (2024). How do institutional gender regimes affect formal reporting processes for sexual harassment? A qualitative study of UK higher education. Law Policy.

[13] Fernando D., Prasad K. (2019). Sex-based harassment and organisational silencing: How women are led to reluctant acquiescence in academia. Hum. Relat..

[14] Hodgins M., Mannix McNamara P. (2019). An Enlightened Environment? Workplace Bullying and Incivility in Irish Higher Education. SAGE Open.

[15] Ferrer J., Holland P., Sinanan R. (2025). Is HRM Missing in Action? A Case Study Approach to Sexual Harassment in Mining. Asia Pac. J. Hum. Resour..

[16] World Health Organization (1986). The Ottawa Charter for Health Promotion.

[17] Mustafa C. (2024). Addressing Willful Blindness: A Multi-Domain Framework for Enhancing Legal Accountability and Fairness. J. Huk. Dan Peradil..

[18] Ballantine C., Fahie D., Hodgins M., MacNeela P., McNamara P.M., McCurtain S., Murphy C. (2026). ‘Heard and Seen and Safe’? Experiences of Disclosing and Reporting Gender-Based and Sexual Violence and Harassment (GBSVH) in Higher Education Institutions in Ireland.

[19] Ahmed S. (2021). Complaint!.

[20] O’Connor P., Hodgins M., Woods D.R., Wallwaey E., Palmen R., Van Den Brink M., Schmidt E.K. (2021). Organisational Characteristics That Facilitate Gender-Based Violence and Harassment in Higher Education?. Adm. Sci..

[21] Agócs C. (1997). Institutionalized Resistance to Organizational Change: Denial, Inaction and Repression. J. Bus. Ethics.

[22] Tauber S. (2022). Women Academics’ Intersectional Experiences of Policy Ineffectiveness in the European Context. Front. Psychol..

[23] Einarsen K., Mykletun R.J., Einarsen S.V., Skogstad A., Salin D. (2017). Ethical Infrastructure and Successful Handling of Workplace Bullying. Nord. J. Work. Life Stud..

[24] Anitha S., Jordan A., Chanamuto N. (2024). The politics of naming and construction: University policies on gender-based violence in the UK. Gend. Educ..

[25] Bull A., Page T. (2022). The Governance of Complaints in UK Higher Education: Critically Examining ‘Remedies’ for Staff Sexual Misconduct. Soc. Leg. Stud..

[26] Bull A. (2025). Exploring Implementation of Gender-Based Violence Policies in English Higher Education. Debate Fem..

[27] Bedera N. (2023). I Can Protect His Future, but She Can’t Be Helped: Himpathy and Hysteria in Administrator Rationalizations of Institutional Betrayal. J. High. Educ..

[28] Shinkwin N. (2021). UniSAFE National Researcher Report Ireland. https://zenodo.org/records/5769121#.YbGvhL3MKUk.

[29] Tsouros A., Tsouros A.D., Dowding G., Thompson J., Dooris M. (1998). From the healthy city to the healthy university: Project development and networking. Health Promoting Universities Concept, Experience and Framework for Action.

[30] (2022). International Labour Organisation Experiences of Violence and Harassment at Work: A Global First Survey.

[31] UniSafe (2022). Results from the Largest European Survey on Gender-Based Violence in Academia. https://unisafe-gbv.eu/project-news/results-from-the-largest-european-survey-on-gender-based-violence-in-academia/.

[32] Humbert A.L., Strid S., Tanwar J., Lipinsky A., Schredl C. (2025). The Role of Intersectionality and Context in Measuring Gender-Based Violence in Universities and Research-Performing Organizations in Europe for the Development of Inclusive Structural Interventions. Violence Against Women.

[33] Kelly L. (1988). Surviving Sexual Violence.

[34] Berlingieri A. (2015). Workplace bullying: Exploring an emerging framework. Work. Employ. Soc..

[35] Harrington S., Warren S., Rayner C. (2015). Human Resource Management practitioners’ responses to workplace bullying: Cycles of symbolic violence. Organization.

[36] Meyer H. (2025). Navigating Workplace Bullying: A Critical Theory Exploration of Lecturers’ Experiences in a Higher Education Context. Hum. Resour. Dev. Q..

[37] McCarry M., Jones C. (2022). The equality paradox: Sexual harassment and gender inequality in a UK university. J. Gend. Stud..

[38] Cortina L., Areguin M.A. (2021). Putting People Down and Pushing Them Out: Sexual Harassment in the Workplace. Annu. Rev. Organ. Psychol. Organ. Behav..

[39] Hearn J., Strid S., Husu L. (2025). Gender-based violence in higher education and research performing organisations: Three steps in critique and reconceptualisation. J. Gend.-Based Violence.

[40] Manuel P., Tang G., Weyand A., James P., Sholzberg M. (2023). Academic bullying in science and medicine: The need for reform. Res. Pract. Thromb. Haemost..

[41] Mahmoudi M., Keashly L. (2021). Filling the space: A framework for coordinated global actions to diminish academic bullying. Angew. Chem. Int. Ed. Engl..

[42] Humbert A.L., Ovesen N., Simonsson A., Strid S., Hearn J., Huck A., Andreska Z., Linková M., Sotirovič V.P., Blažytė G. (2022). UniSAFE Report on the Multi-Level Analysis and Integrated Dataset.

[43] Sotirovic V.P., Blazyte G., Limante A., Tereskinas A., Vaiciuniene R. (2024). What makes academia (un)safe—Experiences, observations, and consequences of gender-based violence in different stages of individual researchers’ careers. Gender-Based Violence and the Law: Global Perspectives and Eastern European Practices.

[44] Sallee M.W., Diaz C.R., Lester J. (2013). Sexual harassment, racist jokes and homophobic slurs: When bullies target identity groups. Workplace Bullying in Higher Education.

[45] Equality and Human Rights Commission (2019). Racial Harassment Inquiry: Survey of Universities.

[46] Phipps A. (2020). Reckoning up: Sexual harassment and violence in the neoliberal university. Gend. Educ..

[47] Hodgins M., Mannix McNamara P. (2017). Bullying and incivility in higher education workplaces: Micropolitics and the abuse of power. Qual. Res. Organ. Manag. Int. J..

[48] Thomas M. (2004). Bullying among support staff in a higher education institution. Health Educ..

[49] Meriläinen M., Sinkkonen H., Puhakka H., Käyhkö K. (2016). Bullying and inappropriate behaviour among faculty personnel. Policy Futur. Educ..

[50] Meriläinen M., Koiv K., Honkanen A. (2019). Bullying effects on performance and engagement among academics. Empl. Relat..

[51] Cullinan J., Hodgins M., Hogan V., Pursell L. (2020). The value of lost productivity from workplace bullying in Ireland. Occup. Med..

[52] Reeves L., Whelan N., Campbell M., Hayes G. (2024). Silenced and Stressed; impact of reporting bullying and harassment for academic staff in Irish higher education. Int. J. Workplace Health Manag..

[53] Humbert A., Strid S. (2025). Institutional confidence, under-reporting and academic consequences of gender-based violence among university staff and students in Europe. Stud. High. Educ..

[54] Burke P.J., Coffey J., Parker J., Hardacre S., Cocuzzoli F., Shaw J., Haro A. (2025). ‘It’s a lot of shame’: Understanding the impact of gender-based violence on higher education access and participation. Teach. High. Educ..

[55] MacNeela P., Dawson K., O’Rourke T., Healy-Cullen S., Burke L., Flack W. (2022). Report on the National Survey of Student Experiences of Sexual Violence and Harassment in Irish Higher Education Institutions.

[56] Mercelle J., Murphy E. (2017). The Neoliberalisation of Irish Higher Education under Austerity. Crit. Sociol..

[57] Hodgins M., McNamara P.M. (2021). The Neoliberal university in Ireland: Institutional bullying by another name?. Societies.

[58] O’Connor P., White K., O’Connor P., White K. (2021). Power, Legitimating Discourses and Institutional Resistance to Gender Equality in Higher Education. Gender, Power and Higher Education in a Globalised World.

[59] Hodgins M., MacCurtain S., Mannix McNamara P. (2020). Power and inaction: Why organizations fail to address workplace bullying. Int. J. Workplace Health Manag..

[60] Ranea-Triviño B., Pajares L., Bustelo M., Pereira B. (2022). Report on the Case Studies on the Effects and Consequences of Institutional Responses to Gender-Based Violence anlong the 7Ps in Research Performing Organisations.

[61] Shannon E.R. (2022). Protecting the Perpetrator: Value Judgements in US and English University Sexual Violence Cases. Gend. Educ..

[62] Keashly L., Neuman J., Lester J. (2013). Bullying in academia: What does current theorizing and research tell us?. Workplace Bullying in Higher Education.

[63] Aukland K. (2023). Sweden Has Surveyed Sexual Harassment in Academia Kifinfo. https://kifinfo.no/en/2022/08/sweden-has-surveyed-sexual-harassment-academia.

[64] Huck A., Andreska Z., Dvořáčková J. (2022). Inventory of Policies and Measures to Respond to GBV in European Universities and Research Organisations.

[65] Thirlwall A. (2015). Organisational sequestering of workplace bullying: Adding insult to injury. J. Manag. Organ..

[66] Hershcovis M.S., Vranjes I., Berdahl J., Cortina L. (2021). See No Evil, Hear No Evil, Speak No Evil: Theorizing Network Silence Around Sexual Harassment. J. Appl. Psychol..

[67] Huck A., Zuzana A., Dvřáčková J., Linková M. (2022). UniSAFE 5.1: Inventory of Policies and Measures to Respond to GBV in European Universities and Research Organisations.

[68] Government of Ireland (2019). Safe, Respectful, Supportive and Positive. Ending Sexual Violence and Harassment in Irish Higher Education Institutions.

[69] Flood M., Dragiewicz M., Pease B. (2020). Resistance and backlash to gender equality. Aust. J. Soc. Policy Issues.

[70] Lukes S. (1974). Power: A Radical View.

[71] Bowen G. (2009). Document Analysis as a Qualitative Research Method. Qual. Res. J..

[72] Sankofa N. (2022). Critical method of document analysis. Int. J. Soc. Res. Methodol..

[73] Health and Safety Authority (2021). Code of Practice for Employers and Employees on the Prevention and Resolution of Bullying at Work.

[74] Rayner C., Lewis D., Einarsen S., Hoel H., Zapf D., Cooper C.L. (2020). Managing workplace bullying: The role of policies. Bullying and Harassment in the Workplace; Theory, Research and Practice.

[75] Richards J., Daley H. (2003). Bullying Policy: Development, Implementation, and Monitoring.

[76] Ranney F. (2000). Beyond Foucault: Toward a User-centered Approach to Sexual Harassment Policy. Techincal Commun. Q..

[77] Ahmed S. (2012). On Being Included: Racism and Diversity in Institutional Life.

[78] Howlett C. (2019). Stop being so melodramatic! or, the problem with sexual harassment policies. Educ. Theory.

[79] Clair R.P. (1993). The bureaucratization, commodification, and privatization of sexual harassment through institutional discourse: A study of the big ten universities. Manag. Commun. Q..

[80] Lewis D. (2004). Bullying at work: The impact of shame among university and college lecturers. Br. J. Guid. Couns..

[81] Cowan S., Munro V.E. (2021). Seeking campus justice: Challenging the ‘criminal justice drift’ in United Kingdom university responses to student violence and misconduct. J. Law Soc..

[82] Sotirovic V.P., Lipinsky A., Struzińska K., Ranea-Triviño B. (2024). You Can Knock on the Doors and Windows of the University, but Nobody Will Care: How Universities Benefit from Network Silence around Gender-Based Violence. Soc. Sci..

[83] Dougherty D.S., Goldstien Hode M. (2016). Binary logics and the discursive interpretation of organizational policy: Making meaning of sexual harassment policy. Hum. Relat..

[84] Martin S., Klein A. (2013). The presumption of mutual influence in occurrences of workplace bullying: Time for change. J. Aggress. Confl. Peace Res..

[85] Government of Ireland (2021). Healthy Ireland at Work: A National Framework for Healthy Workplaces in Ireland.

[86] WHO (2015). Okanagan Charter: An International Charter for Health Promoting Universities and Colleges.

[87] Tauber S., Moughalian C. (2021). Collective system-supporting inaction: A conceptual framework of privilege maintenance. Eur. J. Soc. Psychol..

[88] Bacharach S.B., Baratz M.S. (1962). The two faces of power. Am. Political Sci. Rev..

[89] Sadan E. Theories of Power 2004. http://www.mpow.org/elisheva_sadan_empowerment.pdf.

[90] Matusitz J., Palermo L. (2014). The Disneyfication of the World: A Globalisation Perspective. J. Organ. Transform. Soc. Change.

[91] Rosenthal M., Freyd J. (2022). From DARVO to Distress: College Women’s Contact with their Perpetrators after Sexual Assault, Journal of Aggression. J. Aggress. Maltreatment Trauma.

[92] Yamada D. (2019). Workplace Bullying, DARVO, and Aggressors Claiming Victim Status: Workplace Institute. https://newworkplace.wordpress.com/2019/03/07/workplace-bullying-darvo-and-aggressors-claiming-victim-status/.

[93] Klein A., Martin S. (2011). Two dilemmas in dealing with workplace bullies—False positives and deliberate deceit. Int. J. Workplace Health Manag..

[94] McGlynn C. (2022). Challenging anti-carceral feminism: Criminalisation, justice and continuum thinking. Women’s Stud. Int. Forum.

[95] Woodrow C., Guest D.E. (2013). When good HR gets bad results: Exploring the challenge of HR implementation in the case of workplace bullying. Hum. Resour. Manag. J..

[96] Frasca T., Alvarado I., Buhlmann P., Hutchinson E. (2025). Identifying Gaps in Sexual Harassment Remediation Efforts in Higher Education: Issue Paper.

